# Technology Use Among Caregivers for Persons With and Without Cognitive Impairment

**DOI:** 10.1093/geroni/igaa057.1517

**Published:** 2020-12-16

**Authors:** Shinduk Lee, Matthew Smith, Deborah Vollmer Dahlke, Tiffany Shubert, Steve Popovich, Marcia Ory

**Affiliations:** 1 Texas A&M University, College Station, Texas, United States; 2 DVD Associates, LLC, Austin, Texas, United States; 3 Shubert Consulting, Chapel Hill, North Carolina, United States; 4 Clairvoyant Network, Austin, Texas, United States

## Abstract

This study aims at describing technology use among caregivers for middle-aged or older adults with and without cognitive impairment (CI) and examining whether the associations between technology use, caregiver strain, and social support differ by care-recipient CI status. Online data from caregivers (n=561) for adults at aged 50 years and older were analyzed from a national caregiver and technology survey. Multiple binary items were used to indicate caregivers’ use of various devices (smartphone, computer, e-reader, and wearable activity tracker) and applications (communication, online banking, navigation, online entertainment, medication alert/tracker, and physical activity tracker). Predictors were care-recipient CI status (having been diagnosed with cognitive problems versus no cognitive problems), caregiver strain, and social support. Multivariable logistic regression models were fitted for each technology and for testing effect modification by care- recipient CI status. All models were adjusted for total caregiving hours and caregiver age, sex, race/ethnicity, financial status, and residence. Almost half (47%) reported their care-recipient was diagnosed with CI. Caregivers for those with CI were more likely to use e-readers (adjusted odds ratio (AOR) = 1.55, p=.040), wearable activity trackers (AOR=1.77, p=.013), and medication alerts/trackers (AOR=2.59, p<.001). Generally, greater caregiving strain and social support were positively associated with use of multiple technologies (p<.05). No effect modification of caregiving strain and social support by CI status was observed (p>.05). Technology use differences among caregivers of persons with CI may be driven by care recipients’ unique situations and demands. Future research should identify technology use benefits on caregiver health-related quality of life.

